# Sadness and Loneliness in Adolescents with Physical, Sensory or Health Problems in Low/Middle-Income Countries

**DOI:** 10.3390/children10060996

**Published:** 2023-06-01

**Authors:** Angel Denche-Zamorano, María Ángeles García-Gil, María Mendoza-Muñoz, Sabina Barrios-Fernandez

**Affiliations:** 1 Promoting a Healthy Society (PHeSO) Research Group, Faculty of Sport Sciences, University of Extremadura, 10003 Caceres, Spain; 2 Education Sciences Department, Teacher Training College, University of Extremadura, 10003 Caceres, Spain; 3 Research Group on Physical and Health Literacy and Health-Related Quality of Life (PHYQOL), Faculty of Sport Sciences, University of Extremadura, 10003 Caceres, Spain; 4 Departamento de Desporto e Saúde, Escola de Saúde e Desenvolvimento Humano, Universidade de Évora, 7004-516 Évora, Portugal; 5 Occupation, Participation, Sustainability and Quality of Life (Ability Research Group), Nursing and Occupational Therapy College, University of Extremadura, 10003 Caceres, Spain; sabinabarrios@unex.es

**Keywords:** mental health, depression, anxiety, education, adolescence

## Abstract

Feelings of loneliness and sadness are increasing among the global youth, especially in disadvantaged settings. Young people with disabilities from low-income countries may be at greater risk of experiencing such emotions. This study aims to assess the dependence between difficulties/disability and sadness, loneliness and crying for no reason in young people from low- to middle-income countries and to test the risk of experiencing these emotions in young people with different disabilities versus the overall population. A cross-sectional study was conducted based on the Programme for International Student Assessment for Development survey, with 34,604 participants aged 15 years from seven countries: Cambodia, Ecuador, Guatemala, Honduras, Paraguay, Senegal, and Zambia. Dependent relationships were found between difficulties/disabilities and loneliness, sadness and crying. People with disabilities had a higher prevalence of these emotions. The probability of experiencing sadness, loneliness and crying was higher among people with difficulties/disabilities. Young people with disabilities in low-income countries are at a higher risk of experiencing feelings of loneliness, sadness and crying compared to the general population of the same age.

## 1. Introduction

Loneliness is distress caused by perceived dissatisfaction with the quantity or quality of social relationships [1]. Although loneliness can occur at all ages, it is relatively frequent among young people. While short-term loneliness may be adaptive, causing a reconnection with peers and tightening of social relationships, when it becomes chronic it has adverse effects on young people’s physical and mental health, increasing vulnerability to drug use, poorer sleep quality and higher anxiety or depression levels [2]. Loneliness can also affect students’ academic performance [3].

The consideration of family socioeconomic status as a moderating variable between loneliness and mental health is controversial, as this relationship is always significant [4], and negative correlations have been found between family socioeconomic status and stress levels in children [5]. In this respect, the loneliness prevalence in adolescents with an average age of 15 years in four Caribbean countries with a medium-low economic level was found to be 15.3% [1]. Furthermore, in economically developed European countries such as Italy and Germany, more than 80% of children and adolescents report feeling lonely at least sometimes, and between 3 and 22% report chronic feelings of loneliness [6].

The risk factors for the development of loneliness include social isolation. Thus, the COVID-19 pandemic’s confinement has increased loneliness among young people [2,7], especially those living in disadvantaged districts [8]. COVID-19 has also influenced feelings of sadness [7,9,10] and “feeling like crying all the time” [11]. The same results were obtained in a study of Brazilian adolescents aged 15–18 years, noting that confinement was associated with increased feelings of sadness, anxiety and loneliness in young people [6]. School closure during this period was correlated with increased anxiety and loneliness in adolescents and increased stress, sadness and frustration in children [12].

Children and adolescents with disabilities have higher risks of experiencing psychosocial distress. In this respect, anxiety disorders are common in children with developmental disorders: Disability increases the risk of stigma, discrimination, exclusion and isolation [13]. Disability also affects a person’s quality of life, social relationships and well-being by increasing the risk of loneliness, sadness, depression, fear and anger [14]. Children and adolescents with neurodevelopmental disorders are at risk of loneliness, which in turn is associated with mental health and learning disabilities. In this population, loneliness correlates positively with anxiety and depression and negatively with self-esteem, basic psychological needs and hope [15]. In the case of sensory impairment, blind students aged 7–17 years had a 48.33% prevalence rate of clinical sadness [16] and higher levels of loneliness than those with normal vision [17]. Children with developmental motor coordination disorders are at a higher risk of social isolation, poor social skills and difficulty relating to peers and a higher likelihood of developing depression and anxiety compared to typically developing peers [18]. Adolescents with daily abdominal pain cry more often and feel lonelier and sadder than adolescents with occasional abdominal pain [19].

Given that two of the central symptoms of depression in adolescents are loneliness and sadness and the high association between crying and sadness [20,21]; that the presence of physical or psychological disability increases the risk of feeling lonely, sad and like crying [13,14,15,16,17,18,19]; that controversy exists regarding the importance of family socioeconomic status [4,5,6]; and that most studies have been conducted in developed countries, there is a need for further studies in this regard. This study aimed to assess the relationship between disability and sadness, loneliness and crying for no reason in young people from low- to middle-income countries and to test the risk of experiencing these emotions in young people with different disabilities versus the overall population.

## 2. Materials and Methods

### 2.1. Design and Data

A cross-sectional study was conducted using data from the Programme for International Student Assessment for Development (PISA-D) survey with 34,604 15-year-old participants from seven countries: Cambodia, Ecuador, Guatemala, Honduras, Paraguay, Senegal, and Zambia. This survey has been conducted every three years since 2000 by the Organization for Economic Co-operation and Development (OECD). The survey is internationally standardized and assesses 15-year-old students’ knowledge and skills in more than 80 countries and economies. Its purpose is to compare the extent to which their education systems prepare young people both for their lives and for work [22]. In 2013, several members launched the PISA-D initiative, a pilot exercise to extend PISA to low- and middle-income countries, to provide policymakers from participating countries with information to make their education systems work more effectively, to help develop assessments of learning and to analyze the results to support education policy and decision making [23].

### 2.2. Procedure and Variants 

The PISA-D database was accessed via the OECD website https://www.oecd.org/pisa/pisa-for-development/database/ (accessed on 1 June 2022). Data were then extracted for the following variables: age (from item “Age”, in years); gender (from item “ST004Q01TA”: Are you female or male? Options: Female or Male); country (from item “CNT”: Cambodia, Ecuador, Guatemala, Honduras, Paraguay, Senegal, or Zambia); rural stratum indicator (From item “RuralStr”: Rural Stratum or Urban Stratum); Satisfied_Life (From item “ST015Q01NA”: Overall, how satisfied are you with your life in general these days? Options: From 0 (Not at all satisfied) to 10 (Completely satisfied)). For this research, responses to this item were grouped into: Not at all satisfied (responses from 0 to 4), Satisfied (responses from 5 to 7) and Completely satisfied (responses from 8 to 10); See_Problems (From item “ST016Q01NA”: I can see what is written on the board without difficulty. Options: Yes or No); Hear_Problems (from item “ST016Q02NA”: I can clearly hear the teacher’s voice when he/she is giving a lecture. Options: Yes or No); Walk_Problems (from item “ST016Q03NA”: I have a physical disability that makes it difficult for me to walk or use stairs. Options: Yes or No); Grasp_Problems (from item “ST016Q04NA”: I have a physical disability that makes it difficult for me to grasp small objects such as a pencil or scissors. Options: Yes or No); Sick_Cannot_Play (from item “ST016Q05NA”: I often get so sick that I can’t play, work, or go to school. Options: Yes or No); disability (this was considered as Yes when participants had answered Yes to some of the above items and No when they answered No to all of them); Fun_than_me (from item “ST017Q08NA”: Other students seem to have more fun than me. Options: Never or hardly ever, About once a week, 2 or 3 times a week, or Almost every day). For this study, responses to this item were grouped into: Never (Never or hardly ever), Occasionally (About once a week) or Often (2–3 times a week or Almost every day); Alone (from item “ST017Q07NA”: I feel lonely. Options: Never or hardly ever, About once a week, 2 or 3 times a week or Almost every day. For this study, the responses to this item were grouped into: Never (Never or hardly ever), Occasionally (About once a week) or Often (2–3 times a week or Almost every day)); Sad (from item “ST017Q09NA”: I feel sad or depressed. Options: Never or hardly ever, About once a week, 2 or 3 times a week or Almost every day. For this study, responses to this item were grouped into: Never (Never or hardly ever), Occasionally (About once a week) or Often (2–3 times a week or Almost every day)); and I cry (from item “ST017Q06NA”: I cry for no good reason. Options: Never or hardly ever, About once a week, 2 to 3 times a week, or Almost every day. For this study, the responses to this item were grouped into: Never (Never or hardly ever), Occasionally (About once a week) or Often (2–3 times a week or Almost every day)).

### 2.3. Participants

The PISA-D target population consisted of 15-year-old students (15.25 and 16.33 years) belonging to schools in the seventh grade or above in the countries within the study, including students enrolled full- or part-time in educational institutions, in vocational or other related programs, or foreign schools within the country. Students enrolled at home, in the workplace or outside the country as well as those not enrolled in school were not included. A two-stage stratified sampling scheme was used: (1) for schools with PISA-D eligible students, schools were randomly selected from the full national list of available schools using a systematic probability sampling system proportional to size; and (2) students from these schools were randomly selected from the school’s student list. A target cluster size (TCS) was set for each country. All information on the sample design, sample selection and the technical explanation of the survey development is defined in the PISA-D technical report published by the OECD. Finally, the PISA-D sample was 34,604 participants. Table 1 shows the distribution of participants by gender, country and area of residence, as well as median age [24].

### 2.4. Statistical Analysis

The distribution, followed by the data of the variables, was analyzed with the Kolgomorov–Smirnov test. A descriptive analysis was performed, presenting the sample according to functionality, health status, disability status, life satisfaction and feelings, in absolute and relative frequencies and by gender. A chi-square test was used to analyze the relationships between gender and the variables of interest, and we performed a post hoc pairwise z-test for independent proportions to analyze possible differences in proportions between genders. The same tests were carried out to analyze possible relationships between functional or health problems, disability status and feelings (chi-square test) and possible differences in proportion in these feelings between disabled and non-disabled students according to the different functional or health problems (post-hoc pairwise z-test for independent proportions). The odds ratios (ORs) and their confidence intervals (95% CI) of having negative feelings as a function of having a disability as well as according to each functional or health problem compared to not having a disability were calculated.

## 3. Results

Table S1 displays the descriptive analysis of the sample, with participants being presented according to sensory or health status, life satisfaction and feelings. Gender was related to vision, walking, grasping small objects problems, illness, and disability status (*p* < 0.001). There was a higher proportion of girls with vision issues (18.0% vs. 12.9%, *p* < 0.05), illnesses (15.4% vs. 14.2%, *p* < 0.05) and problems/disabilities (34.9% vs. 30.8%, *p* < 0.05) compared to boys. The proportions of boys with walking (7.7% vs. 6.5%, *p* < 0.05) and grasping small objects problems (8.1% vs. 7.1%, *p* < 0.05) were higher than in girls. Gender was related to life satisfaction (X^2^ = 15.0, df = 2, *p* < 0.001) in students reporting being completely satisfied with their lives, with no differences in proportions between genders. However, differences in proportions were found in those reported being satisfied (higher in girls than boys, 21.7% vs. 20.2%, *p* < 0.05) and dissatisfied (higher in boys than girls, 9.8% vs. 9.0%, *p* < 0.05). Associations were found between gender and feeling like others have more fun (X^2^ = 31.6, df = 2, *p* < 0.001, with a higher proportion of girls than boys (20.3% vs. 18.0%, *p* < 0.05). A higher proportion of girls felt lonely (20.4% vs. 15.4%, *p* < 0.05) or sad (X^2^ = 594.7, df = 2, *p* < 0.001) more frequently than boys. Once again, a higher proportion of girls experienced sadness (21.3% vs. 13.5%, *p* < 0.05)) and crying (X^2^ = 1070.7, df = 2, *p* < 0.05) frequently. The proportion of girls who frequently experienced these feelings were higher than boys (13.6% vs. 5.5%, *p* < 0.05).

**Figure 1:**
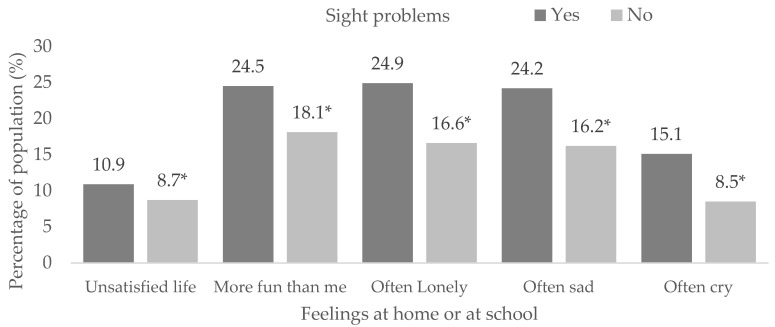
Feelings at home or school in students with sight problems. (*) Significant differences between proportions in the pairwise z-test with *p* < 0.05.

Table S2 shows the associations between sight problems and life satisfaction, more fun than me, loneliness, sadness and crying (*p* < 0.001). There was a lower proportion of students completely satisfied with their lives in those with visual impairment compared to those without it (64.8% vs. 71.0%, *p* < 0.05). A higher proportion of students with sight problems often felt that others had more fun than them (24.5% vs. 18.1%, *p* < 0.05), were lonelier (24.9% vs. 16.6%, *p* < 0.05), sadder (24.2% vs. 16.2%, *p* < 0.05) and cried more (15.1% vs. 8.5%, *p* < 0.05). The proportion of students with life dissatisfaction was higher in those with visual impairments than in those without it (10.9% vs. 8.7%, *p* < 0.05). The proportions of those that never felt that others had more fun, felt lonely, sad, or wanted to cry were higher for students without sight issues than for those with them (Figure 1).

**Figure 2:**
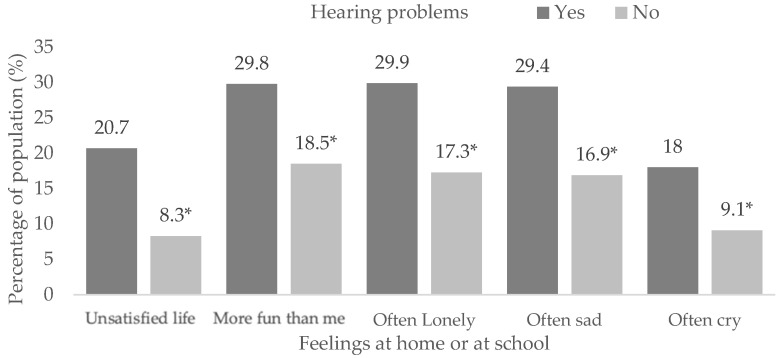
Feelings at home or school in students with hearing problems. (*) Significant differences between proportions in the pairwise z-test with *p* < 0.05.

Similar relationships were found in students with hearing impairments (Table S3) according to life satisfaction (X^2^ = 355.8, df = 2, *p* < 0.001), others have more fun (X^2^ = 129.1, df = 2, *p* < 0.001), loneliness (X^2^ = 184.1, df = 2, *p* < 0.001), sadness (X^2^ = 177.3, df = 2, *p* < 0.001) and crying (X^2^ = 203.3, df = 2, *p* < 0.001), presenting higher proportions of dissatisfaction in life (20.7% vs. 8.3%, *p* < 0.05) and frequent negative feelings (*p* < 0.05) (Figure 2).

**Figure 3:**
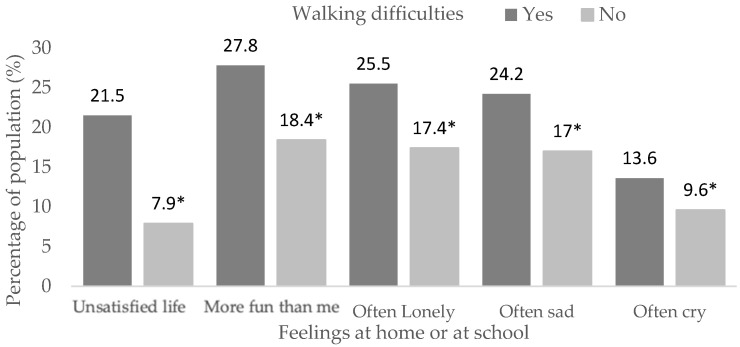
Feelings at home or school in students with walking difficulties. (*) Significant differences between proportions in the pairwise z-test with *p* < 0.05.

Those with walking difficulties (Table S4) had the following values associated with life satisfaction (X^2^ = 491.3, df = 2, *p* < 0.001), others have more fun (X^2^ = 112.1, df = 2, *p* < 0.001), loneliness (X^2^ = 101.6, df = 2, *p* < 0.001), sadness (X^2^ = 86.9, df = 2, *p* < 0.001) and crying (X^2^ = 218.8, df = 2, *p* < 0.001), with higher proportions of dissatisfaction in life (21.5% vs. 7.9%, *p* < 0.05) and having negative feelings frequently (*p* < 0.05) compared to students without this problem (Figure 3).

**Figure 4:**
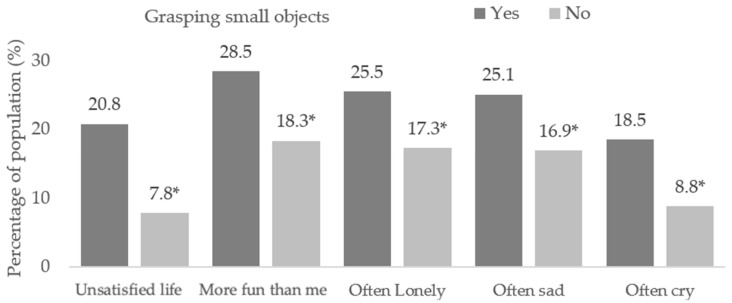
Feelings at home or school students reporting difficulties grasping small objects. (*) Significant differences between proportions in the pairwise z-test with *p* < 0.05.

Those with issues related to grasping small objects (Table S5), obtained the following results in life satisfaction (X^2^ = 459.0, df = 2, *p* < 0.001), others have more fun (X^2^ = 157.4, df = 2, *p* < 0.001), loneliness (X^2^ = 143.1, df = 2, *p* < 0.001), sadness (X^2^ = 112.3, df = 2, *p* < 0.001) and crying (X^2^ = 299.2, df = 2, *p* < 0.001). Higher proportions of dissatisfaction in life (20.8% vs. 7.8%, *p* < 0.05) and having negative feelings more frequently (*p* < 0.05) were found in those with this difficulty than in those without (Figure 4).

**Figure 5:**
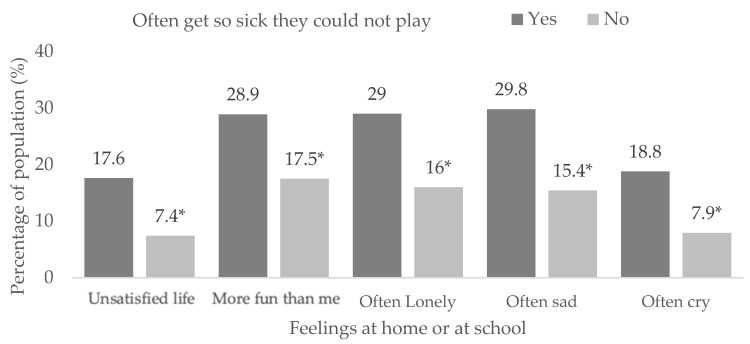
Feelings at home or school in students reporting being often so sick they could not play. (*) Significant differences between proportions in the pairwise z-test with *p* < 0.05.

Among those who reported being so sick that they could not play, the following values were found: life satisfaction (X^2^ = 593.6, df = 2, *p* < 0.001), others have more fun (X^2^ = 355.1, df = 2, *p* < 0.001), loneliness (X^2^ = 517.5, df = 2, *p* < 0.001), sadness (X^2^ = 654.5, df = 2, *p* < 0.001) and crying (X^2^ = 710.0, df = 2, *p* < 0.001) (Table S6). Higher proportions of life dissatisfaction (17.6% vs. 7.4%, *p* < 0.05) and having negative feelings more frequently (*p* < 0.05) were found in those who were sick compared to those who were not (Figure 5).

**Figure 6:**
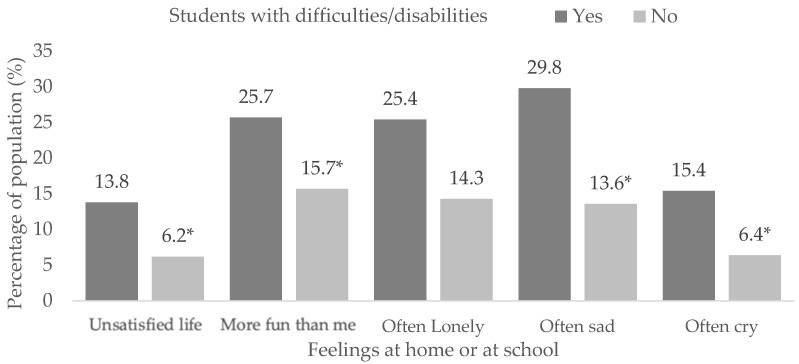
Feelings at home or school in students with difficulties/disabilities. (*) Significant differences between proportions in the pairwise z-test with *p* < 0.05.

The results of the students with difficulties/disabilities in Table S7 show the following scores for life satisfaction (X^2^ = 632.9, df = 2, *p* < 0.001), others have more fun (X^2^ = 482.8, df = 2, *p* < 0.001), loneliness (X^2^ = 671.2, df = 2, *p* < 0.001), sadness (X^2^ = 762.7, df = 2, *p* < 0.001) and crying (X^2^ = 926.6, df = 2, *p* < 0.001). Higher proportions of life dissatisfaction (13.8% vs. 6.2%, *p* < 0.05) and having negative feelings more frequently (*p* < 0.05) were found in those with difficulties/disabilities compared to those who did not (Figure 6).

Increased risks of life dissatisfaction and recurrent negative feelings were found in students with at least one difficulty/disability compared to those without: life dissatisfaction (OR: 2. 63, 95% CI: 2.43–2.85), more fun than me (OR: 1.95, 95% CI: 1.84–2.07), loneliness (OR: 2.20, 95% CI: 2.07–2.34), sadness (OR: 2.36, 95% CI: 2.22–2.51) and crying (OR: 2.88, 95% CI: 2.66–3.11). Similarly, these risks were found to be increased for all students with each of the difficulties/disabilities compared to students without them (Table 2).

## 4. Discussion

Our findings suggest that certain difficulties/disabilities are associated with an increased risk of dissatisfaction with life, recurrent negative feelings, feeling like other people are having more fun, loneliness, sadness and crying for no reason. These differences are maintained in students with difficulties/disabilities even when variables such as gender, income level or parental status are controlled for, suggesting a greater vulnerability to emotional problems among students with disabilities. These results are in line with other findings in the scientific literature, which indicate that having difficulties/disabilities increases a person’s risk of developing loneliness, sadness, depression, fear and anger compared to people without disabilities [14,25]. 

This study found lower proportions of life satisfaction among students with visual impairments or issues compared to those without. Students with sight problems often felt that others had more fun than they did, were sad and cried for no reason, and the proportion of those dissatisfied with life was higher among students with visual impairment. In addition, the proportions of those who never felt that others had more fun, were lonely, sad, or wanted to cry were higher in the non-visual-impairment group. Similar results were obtained for students with hearing problems, difficulties in grasping small objects, and those who often felt sick/ill concerning life satisfaction, the feeling that others have more fun, loneliness, sadness and crying, with students with difficulties/disabilities presenting higher proportions in each of the variables compared to students without those issues. These results highlight the complexity of these variables. Other studies carried out have shown that young people with deafness show similar sadness levels compared to those with normal hearing [26], although they have higher loneliness levels compared to their peers with normal hearing [27]. This could be attributed to discrimination and lack of social acceptance experienced by individuals with deafness, who may feel a sense of isolation [28,29]. A study conducted among adolescents with deafness found that anxiety levels were higher than the average for the overall population [30]. These findings have been confirmed in a study where it was observed that adolescents with deafness had higher levels of anxiety than adolescents without deafness [31]. Other research conducted with deaf young people found no significant differences in levels of sadness compared to levels of sadness in the normal-hearing population [32]. However, despite this, deaf young people reported higher levels of loneliness than their normal-hearing peers. No differences were found between the levels of loneliness of deaf young people who use a cochlear implant and those who do not use a cochlear implant. This suggests that the loneliness experienced by deaf young people is not necessarily the result of a lack of access to auditory communication and that other factors contribute to the loneliness of deaf young people. These factors may include a lack of peer compatibility and understanding, low self-esteem, discrimination and a lack of a sense of belonging [33]. 

On the other hand, the present study shows that students with difficulties/disabilities have higher proportions of difficulties grasping small objects, life satisfaction, others have more fun, loneliness, sadness and crying, compared to students without difficulties/disabilities, in line with other studies that found that children and adolescents with developmental disorders have high levels of loneliness and depression [15]. Similarly, in adolescents with physical disabilities, a prevalence of clinical sadness of 48.33% has been identified in adolescents with sight difficulties [16], and higher levels of loneliness have been identified in these adolescents compared to their peers with normal vision [17]. Higher levels of loneliness and depression are also observed in children with developmental motor coordination problems who are at higher risk of feeling socially isolated and developing anxiety and depression [18]. In contrast, despite the significant correlations between disability and loneliness, sadness and crying, other findings point to a more complex relationship between these factors. Studies by other authors have shown that loneliness is not a consequence of disability alone but is also influenced by other factors such as age, gender, education, marital status, environment and social support [34,35]. 

The results show that students with difficulties/disabilities experience higher levels of life dissatisfaction, the perception that others have more fun, loneliness, sadness and crying relative to students without disabilities. These results are also consistent with other studies that find that adolescents with abdominal pain who often feel sick/are sick [19], cry more frequently and feel lonelier and sadder than adolescents without this symptomatology [19]. Adolescents with cancer experience higher levels of loneliness and isolation compared to their healthy peers [36]. However, another study concludes that loneliness is not a direct consequence of physical or cognitive disability, but instead is influenced by factors such as age, gender, education, environment and social support [18]. In addition, it has been found that people with disabilities have a greater tendency to seek meaningful relationships with others, which allows them to develop abilities to relate to others. This suggests that loneliness in people with disabilities can be managed in part by addressing socio-cultural factors [19]. Adolescents with difficulties/disabilities are more likely to be concerned with life satisfaction, feeling that others are having more fun, loneliness, sadness and crying than students without disabilities, showing that adolescents with disabilities have higher rates of sadness and loneliness than their non-disabled peers. Our results may explain the debate about disability and mental health problems in adolescents by concurring with previous studies showing that people with disabilities are up to five times more likely to suffer from mental, emotional and behavioral disorders than adolescents without disabilities [37]. This may be because disability generates higher levels of psychosocial distress due to the risk of stigma, discrimination, exclusion, and social isolation, affecting the quality of life and social relationships of those affected [13,14]. 

Increased risks of life dissatisfaction and recurrent negative feelings were also found in students with at least one difficulty/disability compared to students with none. However, other work did not find a clear connection between impaired mental health and motor disability [38]; further research is needed to better understand the link between disability and mental health, including a more comprehensive study of the different types of disability, the time course of the condition and the impact of rehabilitation interventions [38].

The practical applications of this research include the importance of developing mental health programs for young people with difficulties/disabilities whose families are of low socio-economic status to ensure that they have support to try and reduce their levels of sadness and crying, thus enabling them to manage the impact of dysfunction/disability on their mental health. These programs could include the assessment of levels of loneliness and the design of strategies to manage them, as well as the promotion of autonomy and social participation of these people. It would also be important to develop education programs for health and education personnel and the families of people with difficulties/disabilities concerning aspects related to loneliness and the improvement of quality of life. These programs could also include the use of technology to improve communication and social contact between people with disabilities and their environment. The development of public policies for the prevention of loneliness in people with difficulties/disabilities with the aim of promoting accessibility and social inclusion is also recommended, both in educational and social settings.

The limitations of this study include the limitations of self-report tests [39]. Another limitation is that this is a correlational study; since it was not possible to manipulate the variables, this does not allow causal inferences to be drawn between the variables [40,41]. In addition, the study design does not allow causal relations to be drawn between the variables. Another limitation is selection bias, which could be a major problem, considering that students with developmental disabilities are less likely to participate in this type of survey. Reversing the data would provide an interesting perspective on protective factors, even though these data represent only a significant part of the sample and not all participants. This situation poses both a limitation in terms of representativeness and an opportunity for future research. In terms of limitation, the results could not be generalized due to the lack of complete representation of the sample. However, this discrepancy can also be seen as an idea for future research since it could indicate the presence of previously unconsidered protective factors. This encourages further exploration of the influence of these factors and encourages further research in the future. 

## 5. Conclusions

This study aimed to assess the correlation between the presence of difficulties/disabilities with sadness, loneliness and crying in adolescents from low- and middle-income countries and to compare the risk of developing this symptomatology in a variety of young people with disabling conditions compared to young people without disabilities. The correlation between disability and sadness, loneliness and crying was found to be significant, and there is a higher prevalence of sadness, loneliness and crying among young people with difficulties/disabilities than among young people without them.

## Figures and Tables

**Table 1:** Socio-demographic characteristics of participants.

	Overall = 34,604		Men = 16,796		Women = 17,808	
Variables	Median	IQR	Median	IQR	Median	IQR
Age (Years)	15.8	(0.5)	15.8	(0.5)	15.8	(0.5)
Country	n	%	n	%	n	%
Cambodia	5162	14.9	2434	47.2	2728	52.8
Ecuador	5664	16.4	2887	51.0	2777	49.0
Guatemala	5100	14.7	2641	51.8	2459	48.2
Honduras	4773	13.8	2216	46.4	2557	53.6
Paraguay	4510	13.0	2219	49.2	2291	50.8
Senegal	5182	15.0	2385	46.0	2797	54.0
Zambia	4213	12.2	2014	47.8	2199	52.2
Rural/Urban						
Urban	21,659	62.6	10,321	47.7	11,338	52.3
Rural	12,945	37.4	6475	50.0	6470	50.0

n: participants; %: percentage; IQR: interquartile range.

**Table 2:** Risks of being dissatisfied in life, having more fun than me, loneliness, sadness and crying according to student difficulty/disability status.

Students with Difficulties and/or Disabilities				
	No	Yes		
Variables		OR	CI 95%	
Dissatisfied in life	Ref.	2.63 *	2.43	2.85
More fun than me	Ref.	1.95 *	1.84	2.07
Loneliness	Ref.	2.20 *	2.07	2.34
Sadness	Ref.	2.36 *	2.22	2.51
Crying	Ref.	2.88*	2.66	3.11
Sight problems				
Dissatisfied in life	Ref.	1.38 *	1.25	1.52
More fun than me	Ref.	1.53 *	1.42	1.65
Loneliness	Ref.	1.72 *	1.60	1.85
Sadness	Ref.	1.79 *	1.66	1.93
Crying	Ref.	2.07 *	1.90	2.27
Hearing issues				
Dissatisfied in life	Ref.	3.37 *	2.94	3.86
More fun than me	Ref.	1.97 *	1.74	2.22
Loneliness	Ref.	2.26 *	2.00	2.55
Sadness	Ref.	2.28 *	2.01	2.58
Crying	Ref.	2.42 *	2.10	2.78
Walking problems				
Dissatisfied in life	Ref.	3.49 *	3.11	3.92
More fun than me	Ref.	1.76 *	1.58	1.95
Loneliness	Ref.	1.72 *	1.55	1.92
Sadness	Ref.	1.68 *	1.50	1.88
Crying	Ref.	2.33 *	2.06	2.63
Grasping small objects problems				
Dissatisfied in life	Ref.	3.28 *	2.93	3.68
More fun than me	Ref.	1.81 *	1.63	2.01
Loneliness	Ref.	1.77 *	1.59	1.97
Sadness	Ref.	1.77 *	1.59	1.97
Crying	Ref.	2.55 *	2.27	2.86
Often getting sick				
Dissatisfied in life	Ref.	2.95 *	2.70	3.24
More fun than me	Ref.	2.04 *	1.89	2.20
Loneliness	Ref.	2.36 *	2.18	2.54
Sadness	Ref.	2.66 *	2.47	2.88
Crying	Ref.	2.94 *	2.69	3.21

OR: odds ratio, OR > 1 indicating a higher risk of reporting the following: dissatisfied in life; more fun than me; loneliness; sadness; crying without a good reason; CI 95%: 95% confidence interval of the odds ratio; *: *p* value < 0.05; Ref.: reference.

## Data Availability

Datasets are available under reasonable request.

## Supplementary Materials

The following supporting information can be downloaded at: https://www.mdpi.com/article/10.3390/children10060996/s1, Table S1: Descriptive analysis of participants according to self-perceived physical, sensory or health problems at school, relationships, and comparisons by gender; Table S2: Descriptive analysis: Feelings.; Table S3: Feelings and sight; Table S4: Feelings and hearing; Table S5: Feelings and walking; Table S6: Feelings and grasping; Table S7: Feelings and being sick; Table S8: Feelings and some problems.
