# Supplementary material for: Sadness and Loneliness in Adolescents with Physical, Sensory or Health Problems in Low/Middle-Income Countries
Source: Children (Basel). 2023 Jun 1;10(6):996. doi: 10.3390/children10060996 (PMC10296925; doi:10.3390/children10060996)
Supplement: Supplementary file 1 [file children-10-00996-s001.zip › children-2405976-supplementary.pdf]

Table S1. Descriptive analysis of participants according to self-perceived physical, sensory or health problems at school, relationships and comparisons by sex.

Variables	Overall = 33301		Men = 16198		Women = 17103				
See_Problems	n	%	n	%	n	%	x^2	df	p
Yes	5176	15.5	2095	12.9	3081	18.0*	163.6	1	<0.001
No	28125	84.5	14103	87.1	14022	82.0*			
Hear_Problems	Overall = 33126		Men = 16083		Women = 17043				
Yes	1644	5.0	789	4.9	855	5.0	0.2	1	0.642
No	31482	95.0	15294	95.1	16188	95.0			
Walk_Problems	Overall = 32502		Men = 15802		Women = 16700				
Yes	2301	7.1	1217	7.7	1084	6.5*	18.1	1	<0.001
No	30201	92.9	14585	92.3	15616	93.5*			
Grasp_Problems	Overall = 32503		Men = 15825		Women = 16678				
Yes	2464	7.6	1276	8.1	1188	7.1*	10.2	1	0.001
No	30039	92.4	14549	91.9	15490	92.9*			
Sick_Cannot_Play	Overall = 32691		Men = 15890		Women = 16801				
Yes	4851	14.8	2261	14.2	2590	15.4*	9.1	1	<0.001
No	27840	85.2	13629	85.8	14211	84.6*			
Some_Problem	Overall = 31873		Men = 15533		Women = 16340				
Yes	10485	32.9	4784	30.8	5701	34.9*	60.4	1	<0.001
No	21388	67.1	10749	69.2	10639	65.1*			

n: Participants; %: Percentage; x^2: Pearson's Chi-square; dl: Degree freedom; p: p-value from Chi-square test; *: Significant differences between sex ratios.

Table S2. Descriptive analysis: Feelings.

Variables	Overall = 33301		Men = 16198		Women = 17103		x^2	df	p
	n	%	n	%	n	%			
Satisfied_Life	Overall = 33163		Men = 16098		Women = 17065				
Not at all	3115	9.4	1577	9.8	19248	9.0*	15.0	2	0.001
Satisfied	6959	21.0	3253	20.2	4963	21.7*			
Completely	23089	69.6	11268	70.0	5100	69.3			
Fun than me	Overall = 32285		Men = 15685		Women = 16600				
Never	20366	63.1	9982	63.6	10384	62.6*	31.6	2	0.001
Occasionally	5733	17.8	2886	18.4	2847	17.2*			
Often	6186	19.2	2817	18.0	3369	20.3*			
Lonely	Overall = 32224		Men = 15634		Women = 16590				
Never	20921	64.9	10774	68.9	10147	61.2*	223.4	2	0.001
Occasionally	5509	17.1	2457	15.7	3052	18.4*			
Often	5594	18.0	2403	15.4	3391	20.4*			
Sad	Overall = 32184		Men = 15607		Women = 16577				
Never	19484	60.5	10485	67.2	8999	54.3*	594.7	2	0.001
Occasionally	7062	21.9	3014	19.3	4048	24.4*			
Often	5638	17.5	2108	13.5	3530	21.3*			
Cry	Overall = 32276		Men = 15661		Women = 16615				
Never	25969	80.5	13756	87.8	12213	73.5*	1070.7	2	0.001
Occasionally	3200	9.9	1050	6.7	2150	12.9*			
Often	3107	9.6	855	5.5	2252	13.6*			

n: Participants; %: Percentage; x^2: Pearson's Chi-square; dl: Degree freedom; p: p-value from Chi-square test; *: Significant differences between sex ratios.

Table S3: Feelings and sight.

Often you fell	I can see what is written on the board without difficulty.						
	No		Yes				
Satisfied life	n	(%)	n	(%)	x^2	df	p
Not at all	553	10.9	2373	8.7*			
Satisfied	1223	24.2	5543	20.3*	77.8	2	<0.001
Completely	3276	64.8	19373	71.0*			
Fun than me							
Never	2825	57.1	17276	64.3*			
Occasionally	909	18.4	4744	17.7	126.6	2	<0.001
Often	1213	24.5	4855	18.1*			
Lonely							
Never	2819	57.2	17842	66.5*			
Occasionally	886	18.0	4540	16.9	217.9	2	<0.001
Often	1227	24.9	4456	16.6*			
Sad							
Never	2572	52.1	16659	62.2*			
Occasionally	1172	23.7	5803	21.7*	233.7	2	<0.001
Often	1197	24.2	4334	16.2*			
Cry without reason							
Never	3482	70.9	22160	82.3*			
Occasionally	684	13.9	2472	9.2*	355.8	2	<0.001
Often	743	15.1	2279	8.5*			

n: Participants; %: Percentage; x^2: Pearson's Chi-square; dl: Degree freedom; p: p-value from Chi-square test; *: Significant differences between see problems status ratio.

Table S4. Feelings and hearing.

Often you fell	I can hear the teacher’s voice clearly when he or she is giving a lesson.						
	No		Yes				
Satisfied_life	n	(%)	n	(%)	x^2	df	p
Not at all	324	20.7	2539	8.3*			
Satisfied	419	26.8	6311	20.6*	355.8	2	<0.001
Completely	823	52.6	21725	71.1*			
Fun than me							
Never	766	51.6	19307	63.8*			
Occasionally	276	18.6	5353	17.7	129.1	2	<0.001
Often	443	29.8	5605	18.5*			
Lonely							
Never	751	50.4	19891	65.8*			
Occasionally	294	19.7	5111	16.9*	184.1	2	<0.001
Often	445	29.9	5225	17.3*			
Sad							
Never	692	46.9	18510	61.3*			
Occasionally	351	23.8	6600	21.9*	177.3	2	<0.001
Often	434	29.4	5093	16.9*			
Cry without reason							
Never	998	66.8	24603	81.3*			
Occasionally	227	15.2	2904	9.6*	202.3	2	<0.001
Often	269	18.0	2743	9.1*			

n: Participants; %: Percentage; x2: Pearson's Chi-square; dl: Degree freedom; p: p-value from Chi-square test; *: Significant differences between see problems status ratio.

Table S5. Feelings and walking.

Often you fell	I have a physical disability that makes it difficult for me to walk or use stairs.						
	Yes		No				
Satisfied_life	n	(%)	n	(%)	x^2	df	p
Not at all	458	21.5	2330	7.9*			
Satisfied	490	23	6118	20.8*	491.3	2	<0.001
Completely	1181	55.5	20993	71.3*			
Fun than me							
Never	1116	54.7	18723	63.8*			
Occasionally	357	17.5	5227	17.8	112.1	2	<0.001
Often	566	27.8	5408	18.4*			
Lonely							
Never	1137	55.8	19248	65.7*			
Occasionally	383	18.8	4963	16.9*	101.6	2	<0.001
Often	519	25.5	5100	17.4*			
Sad							
Never	1054	51.9	17919	61.1*			
Occasionally	484	23.9	6417	21.9*	86.9	2	<0.001
Often	491	24.2	4970	17.0*			
Cry without reason							
Never	1416	68.9	23909	81.5*			
Occasionally	279	13.6	2827	9.6*	218.8	2	<0.001
Often	360	17.5	2614	8.9*			

n: Participants; %: Percentage; x^2: Pearson's Chi-square; dl: Degree freedom; p: p-value from Chi-square test; *: Significant differences between see problems status ratio.

Table S6. Feelings and grasping.

Often you fell	I have a physical disability that makes it difficult for me to grasp small objects like a pencil or scissors.						
	Yes		No				
Satisfied_life	n	(%)	n	(%)	x^2	df	p
Not at all	471	20.8	2302	7.8*			
Satisfied	488	21.6	6109	20.8	459.0	2	<0.001
Completely	1304	57.6	20920	71.3*			
Fun than me							
Never	1144	52.3	18730	64.0*			
Occasionally	419	19.2	5167	17.7	157.4	2	<0.001
Often	624	28.5	5369	18.3*			
Lonely							
Never	1183	53.7	19251	65.9*			
Occasionally	458	20.8	4901	16.8*	143.1	2	<0.001
Often	562	25.5	5058	17.3*			
Sad							
Never	1126	51.6	17893	61.2*			
Occasionally	507	23.2	6403	21.9	112.3	2	<0.001
Often	548	25.1	4924	16.9*			
Cry without reason							
Never	1485	67.3	23870	81.7*			
Occasionally	313	14.2	2794	9.6*	299.2	2	<0.001
Often	407	18.5	2569	8.8*			

n: Participants; %: Percentage; x^2: Pearson's Chi-square; dl: Degree freedom; p: p-value from Chi-square test; *: Significant differences between see problems status ratio.

Table S7. Feelings and getting sick.

Often you fell	I often get so sick I cannot play, work or go to school.						
	Yes		No				
Satisfied_life	n	(%)	n	(%)	x^2	df	p
Not at all	795	17.6	2015	7.4*			
Satisfied	1099	24.3	5543	20.4*	593.6	2	<0.001
Completely	2628	58.1	19677	72.2*			
Fun than me							
Never	2340	52.7	17603	64.9*			
Occasionally	817	18.4	4794	17.7	355.1	2	<0.001
Often	1286	28.9	4738	17.5*			
Lonely							
Never	2292	51.7	18225	67.3*			
Occasionally	856	19.3	4519	16.7*	517.5	2	<0.001
Often	1288	29.0	4348	16.0*			
Sad							
Never	2026	45.8	17073	63.0*			
Occasionally	1082	24.4	5839	21.6*	654.5	2	<0.001
Often	1320	29.8	4174	15.4*			
Cry without reason							
Never	2968	66.7	22468	82.9*			
Occasionally	643	14.5	2472	9.1*	711.0	2	<0.001
Often	837	18.8	2155	7.9*			

n: Participants; %: Percentage; x2: Pearson's Chi-square; dl: Degree freedom; p: p-value from Chi-square test; *: Significant differences between see problems status ratio.

Table S8. Feelings and some problems.

Often you fell	Some problems …						
	Yes		No				
Satisfied_life	n	(%)	n	(%)	x^2	df	p
Not at all	1383	13.8	1303	6.2*			
Satisfied	2371	23.6	4107	19.6*	632.9	2	<0.001
Completely	6281	62.6	15571	74.2*			
Fun than me							
Never	5594	56.1	13986	66.7*			
Occasionally	1812	18.2	3677	17.5	482.8	2	<0.001
Often	2568	25.7	3290	15.7*			
Lonely							
Never	5581	56	14534	69.4*			
Occasionally	1854	18.6	3414	16.3*	671.2	2	<0.001
Often	2525	25.4	2988.0	14.3*			
Sad							
Never	5077	45.8	13650	65.2*			
Occasionally	2363	24.4	4421	21.1*	762.7	2	<0.001
Often	2505	29.8	2854	13.6*			
Cry without reason							
Never	7106	71.3	17883	85.3*			
Occasionally	1327	13.3	1733	8.3*	926.6	2	<0.001
Often	1540	15.4	1348	6.4*			

n: Participants; %: Percentage; x^2: Pearson's Chi-square; dl: Degree freedom; p: p-value from Chi-square test; *: Significant differences between see problems status ratio.
